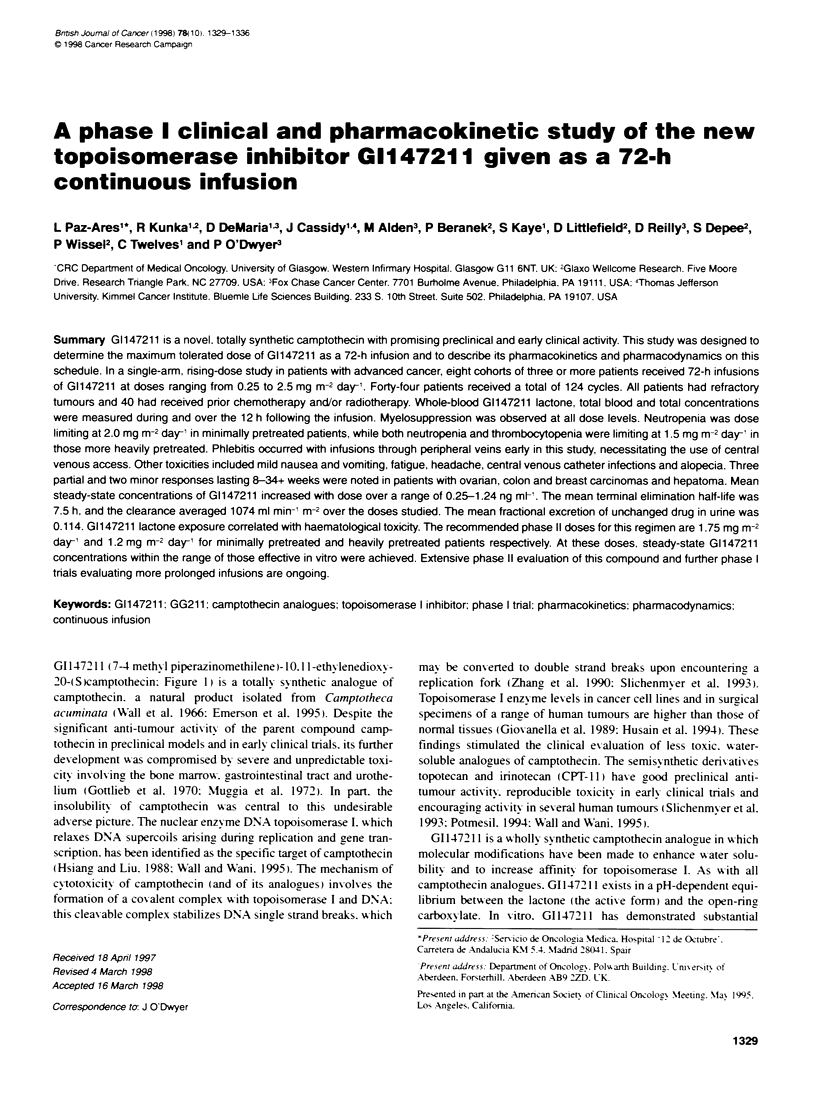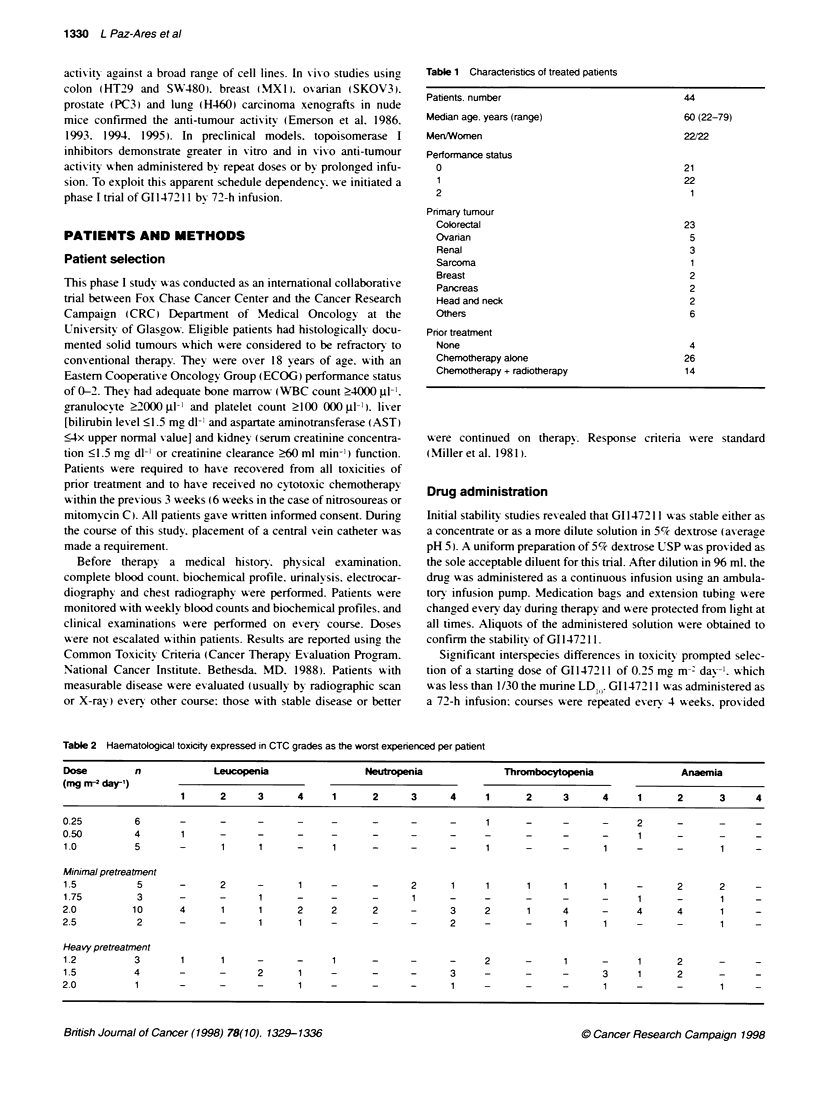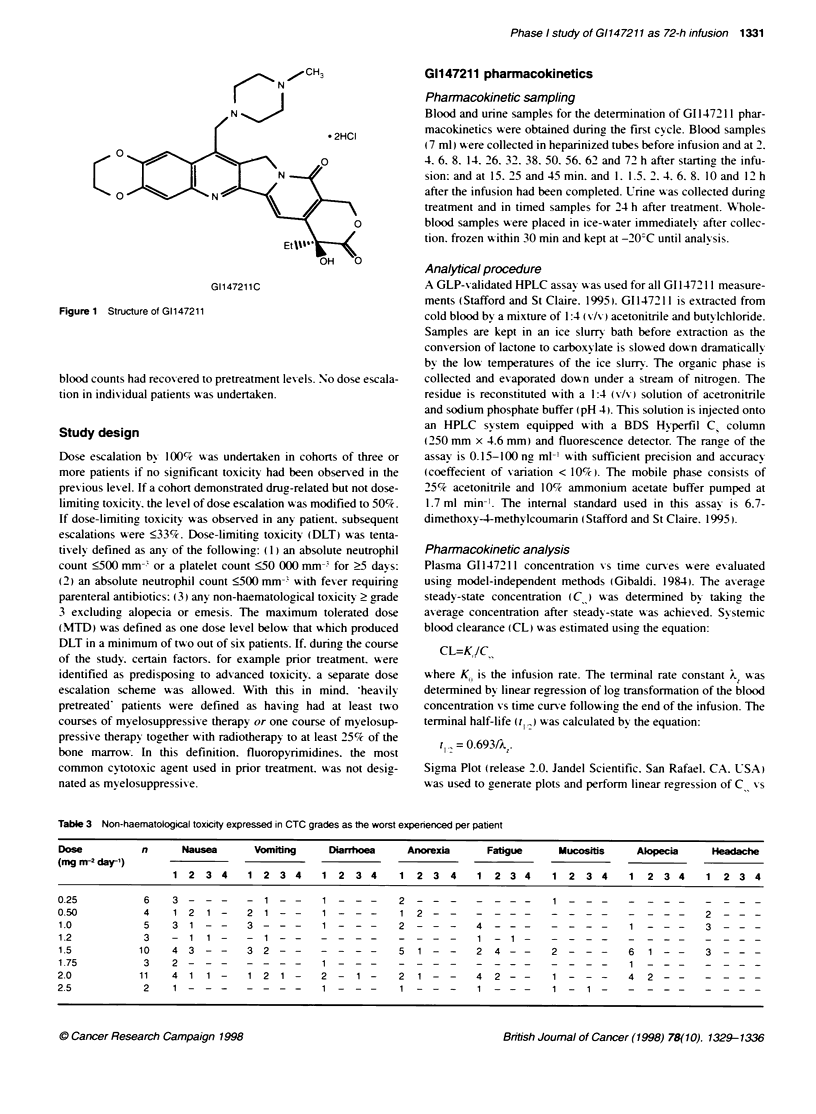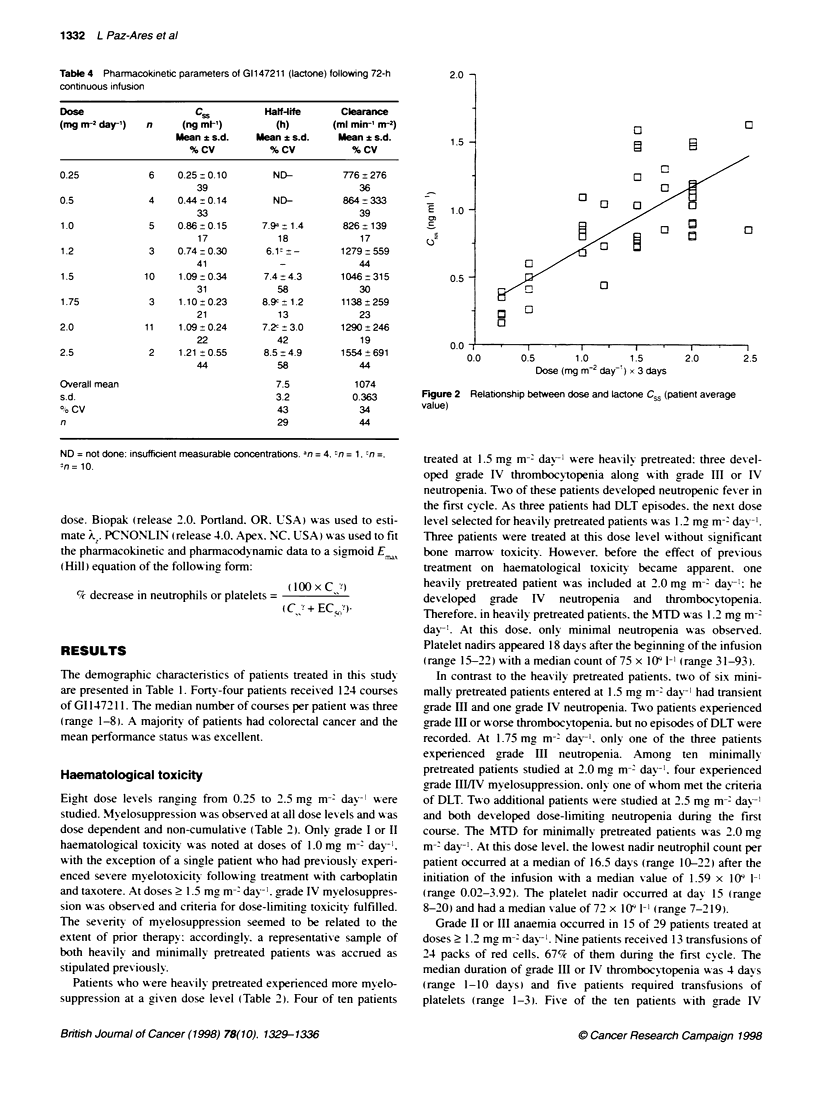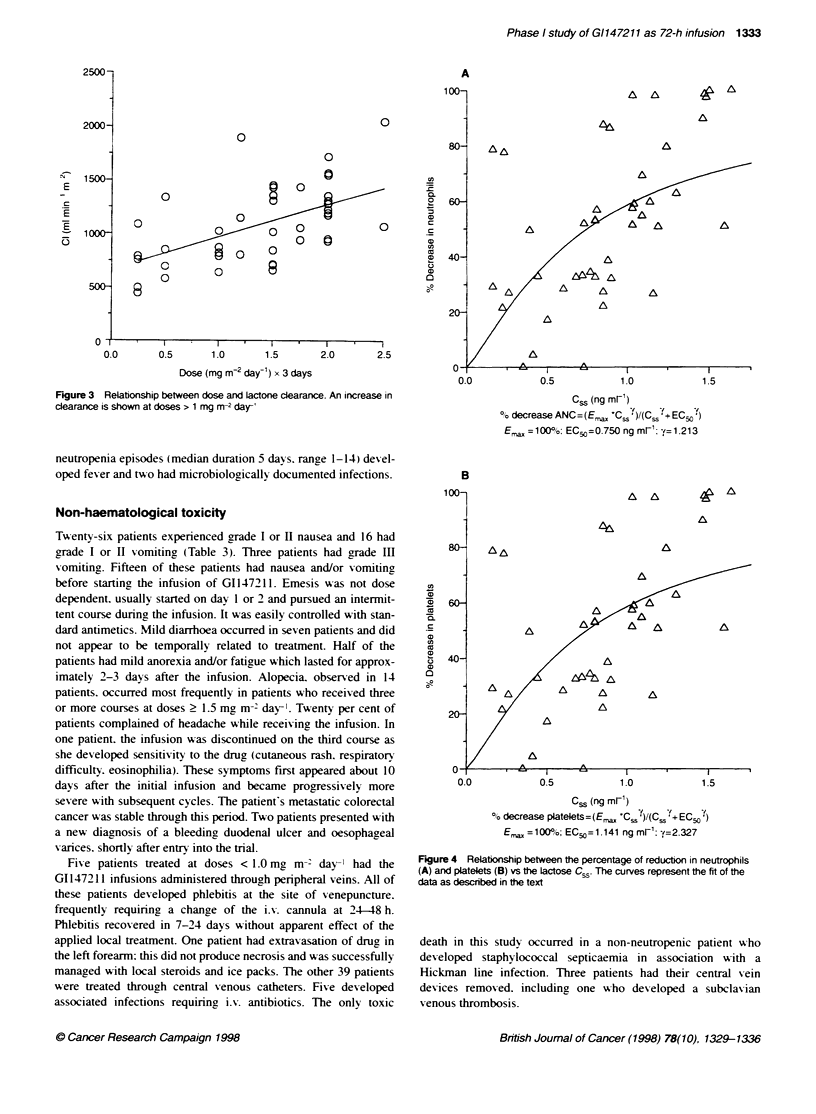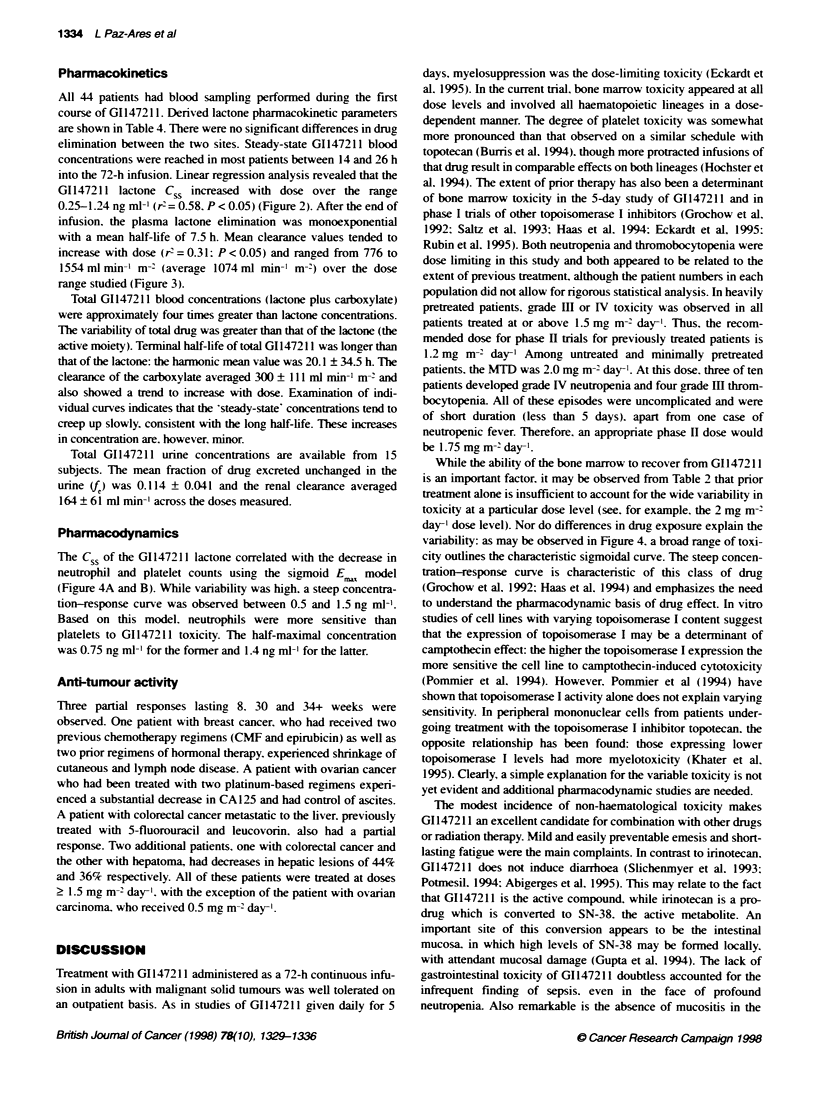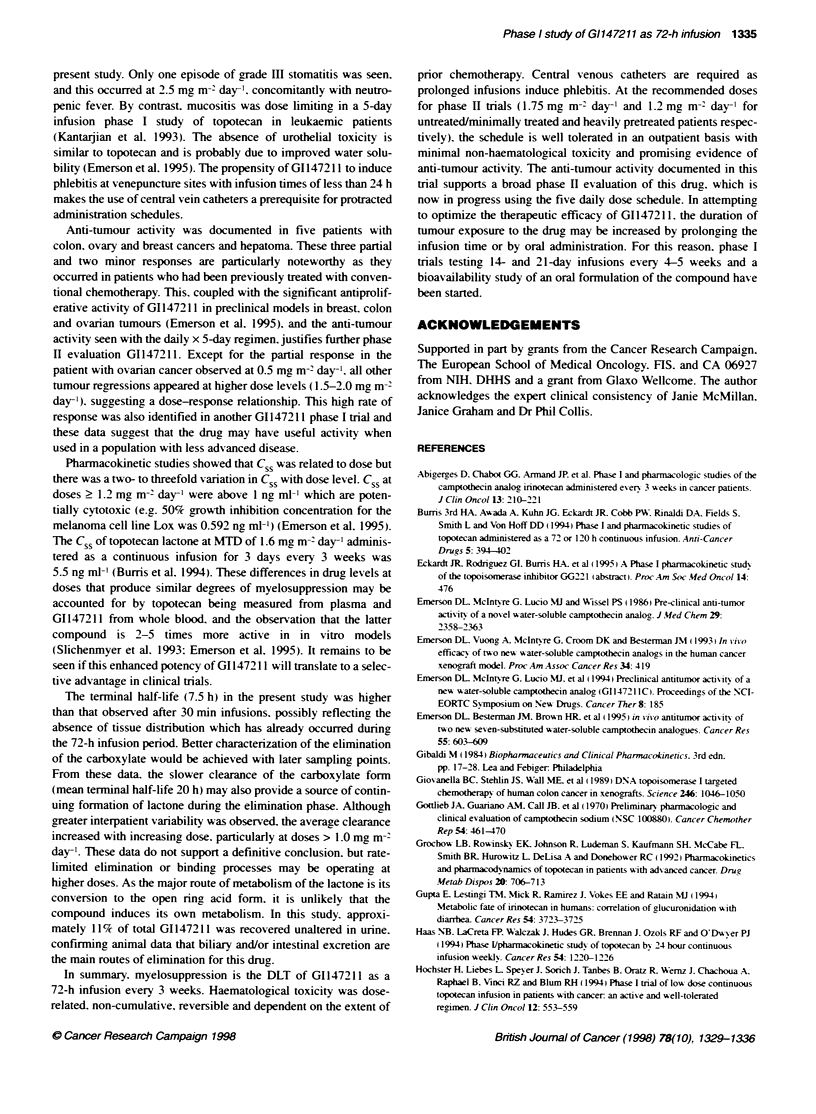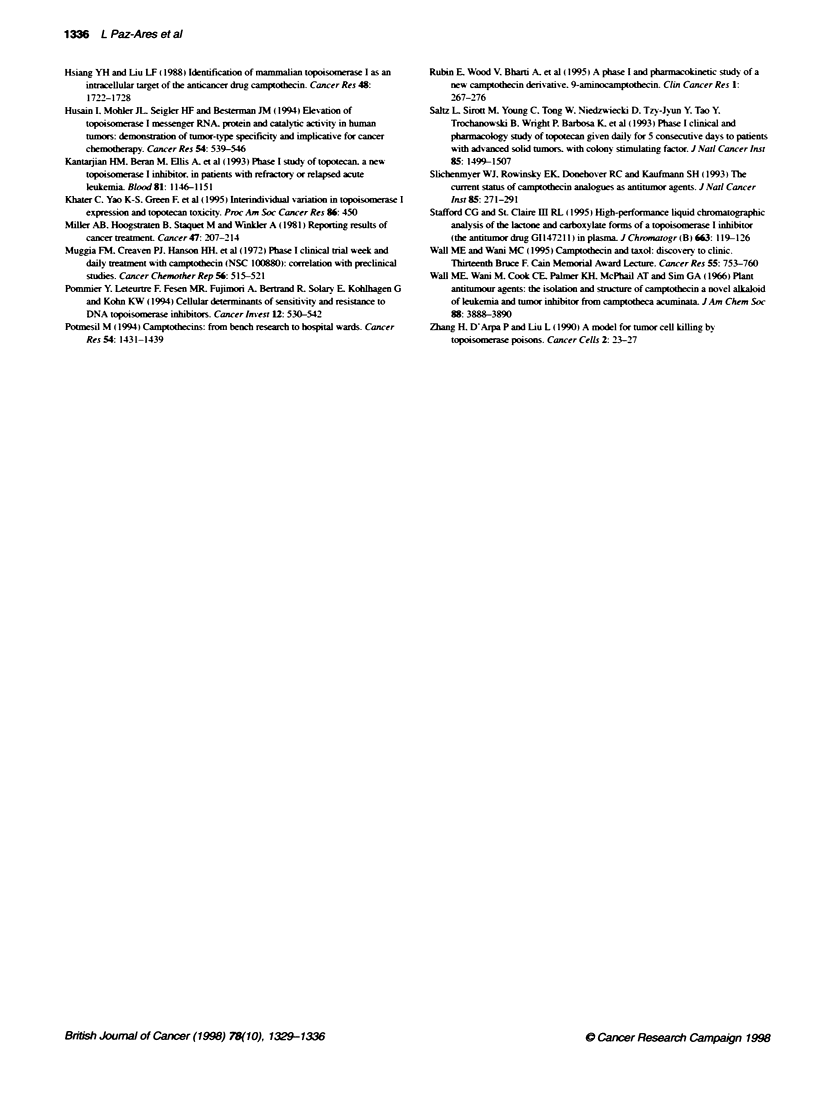# A phase I clinical and pharmacokinetic study of the new topoisomerase inhibitor GI147211 given as a 72-h continuous infusion.

**DOI:** 10.1038/bjc.1998.679

**Published:** 1998-11

**Authors:** L. Paz-Ares, R. Kunka, D. DeMaria, J. Cassidy, M. Alden, P. Beranek, S. Kaye, D. Littlefield, D. Reilly, S. Depee, P. Wissel, C. Twelves, P. O'Dwyer

**Affiliations:** CRC Department of Medical Oncology, University of Glasgow, Western Infirmary Hospital, UK.

## Abstract

GI147211 is a novel, totally synthetic camptothecin with promising preclinical and early clinical activity. This study was designed to determine the maximum tolerated dose of Gl147211 as a 72-h infusion and to describe its pharmacokinetics and pharmacodynamics on this schedule. In a single-arm, rising-dose study in patients with advanced cancer, eight cohorts of three or more patients received 72-h infusions of Gl147211 at doses ranging from 0.25 to 2.5 mg m(-2) day(-1). Forty-four patients received a total of 124 cycles. All patients had refractory tumours and 40 had received prior chemotherapy and/or radiotherapy. Whole-blood Gl147211 lactone, total blood and total concentrations were measured during and over the 12 h following the infusion. Myelosuppression was observed at all dose levels. Neutropenia was dose limiting at 2.0 mg m(-2) day(-1) in minimally pretreated patients, while both neutropenia and thrombocytopenia were limiting at 1.5 mg m(-2) day(-1) in those more heavily pretreated. Phlebitis occurred with infusions through peripheral veins early in this study, necessitating the use of central venous access. Other toxicities included mild nausea and vomiting, fatigue, headache, central venous catheter infections and alopecia. Three partial and two minor responses lasting 8-34+ weeks were noted in patients with ovarian, colon and breast carcinomas and hepatoma. Mean steady-state concentrations of Gl147211 increased with dose over a range of 0.25-1.24 ng ml(-1). The mean terminal elimination half-life was 7.5 h, and the clearance averaged 1074 ml min(-1) m(-2) over the doses studied. The mean fractional excretion of unchanged drug in urine was 0.114. Gl147211 lactone exposure correlated with haematological toxicity. The recommended phase II doses for this regimen are 1.75 mg m(-2) day(-1) and 1.2 mg m(-2) day(-1) for minimally pretreated and heavily pretreated patients respectively. At these doses, steady-state Gl147211 concentrations within the range of those effective in vitro were achieved. Extensive phase II evaluation of this compound and further phase I trials evaluating more prolonged infusions are ongoing.


British Journal of Cancer (1998) 78(10), 1329–1336
© 1998 Cancer Research Campaign

### A phase I clinical and pharmacokinetic study of the new topoisomerase inhibitor GI147211 given as a 72-h continuous infusion

L Paz-Ares[1*], R Kunka[1,2], D DeMaria[1,3], J Cassidy[1,4], M Alden[3], P Beranek[2], S Kaye[1], D Littlefield[2], D Reilly[3], S Depee[2], P Wissel[2], C Twelves[1] and P O'Dwyer[3]

[1]CRC Department of Medical Oncology, University of Glasgow, Western Infirmary Hospital, Glasgow G11 6NT, UK; [2]Glaxo Wellcome Research, Five Moore Drive, Research Triangle Park, NC 27709, USA; [3]Fox Chase Cancer Center, 7701 Burholme Avenue, Philadelphia, PA 19111, USA; [4]Thomas Jefferson University, Kimmel Cancer Institute, Bluemle Life Sciences Building, 233 S. 10th Street, Suite 502, Philadelphia, PA 19107, USA

**Summary** GI147211 is a novel, totally synthetic camptothecin with promising preclinical and early clinical activity. This study was designed to determine the maximum tolerated dose of GI147211 as a 72-h infusion and to describe its pharmacokinetics and pharmacodynamics on this schedule. In a single-arm, rising-dose study in patients with advanced cancer, eight cohorts of three or more patients received 72-h infusions of GI147211 at doses ranging from 0.25 to 2.5 mg m$^{-2}$ day$^{-1}$. Forty-four patients received a total of 124 cycles. All patients had refractory tumours and 40 had received prior chemotherapy and/or radiotherapy. Whole-blood GI147211 lactone, total blood and total concentrations were measured during and over the 12 h following the infusion. Myelosuppression was observed at all dose levels. Neutropenia was dose limiting at 2.0 mg m$^{-2}$ day$^{-1}$ in minimally pretreated patients, while both neutropenia and thrombocytopenia were limiting at 1.5 mg m$^{-2}$ day$^{-1}$ in those more heavily pretreated. Phlebitis occurred with infusions through peripheral veins early in this study, necessitating the use of central venous access. Other toxicities included mild nausea and vomiting, fatigue, headache, central venous catheter infections and alopecia. Three partial and two minor responses lasting 8–34+ weeks were noted in patients with ovarian, colon and breast carcinomas and hepatoma. Mean steady-state concentrations of GI147211 increased with dose over a range of 0.25–1.24 ng ml$^{-1}$. The mean terminal elimination half-life was 7.5 h, and the clearance averaged 1074 ml min$^{-1}$ m$^{-2}$ over the doses studied. The mean fractional excretion of unchanged drug in urine was 0.114. GI147211 lactone exposure correlated with haematological toxicity. The recommended phase II doses for this regimen are 1.75 mg m$^{-2}$ day$^{-1}$ and 1.2 mg m$^{-2}$ day$^{-1}$ for minimally pretreated and heavily pretreated patients respectively. At these doses, steady-state GI147211 concentrations within the range of those effective in vitro were achieved. Extensive phase II evaluation of this compound and further phase I trials evaluating more prolonged infusions are ongoing.

**Keywords:** GI147211; GG211; camptothecin analogues; topoisomerase I inhibitor; phase I trial; pharmacokinetics; pharmacodynamics; continuous infusion

GI147211 (7-4 methyl piperazinomethilene)-10,11-ethylenedioxy-20-(S)camptothecin; Figure 1) is a totally synthetic analogue of camptothecin, a natural product isolated from *Camptotheca acuminata* (Wall et al, 1966; Emerson et al, 1995). Despite the significant anti-tumour activity of the parent compound camptothecin in preclinical models and in early clinical trials, its further development was compromised by severe and unpredictable toxicity involving the bone marrow, gastrointestinal tract and urothelium (Gottlieb et al, 1970; Muggia et al, 1972). In part, the insolubility of camptothecin was central to this undesirable adverse picture. The nuclear enzyme DNA topoisomerase I, which relaxes DNA supercoils arising during replication and gene transcription, has been identified as the specific target of camptothecin (Hsiang and Liu, 1988; Wall and Wani, 1995). The mechanism of cytotoxicity of camptothecin (and of its analogues) involves the formation of a covalent complex with topoisomerase I and DNA; this cleavable complex stabilizes DNA single strand breaks, which may be converted to double strand breaks upon encountering a replication fork (Zhang et al, 1990; Slichenmyer et al, 1993). Topoisomerase I enzyme levels in cancer cell lines and in surgical specimens of a range of human tumours are higher than those of normal tissues (Giovanella et al, 1989; Husain et al, 1994). These findings stimulated the clinical evaluation of less toxic, water-soluble analogues of camptothecin. The semisynthetic derivatives topotecan and irinotecan (CPT-11) have good preclinical anti-tumour activity, reproducible toxicity in early clinical trials and encouraging activity in several human tumours (Slichenmyer et al, 1993; Potmesil, 1994; Wall and Wani, 1995).

GI147211 is a wholly synthetic camptothecin analogue in which molecular modifications have been made to enhance water solubility and to increase affinity for topoisomerase I. As with all camptothecin analogues, GI147211 exists in a pH-dependent equilibrium between the lactone (the active form) and the open-ring carboxylate. In vitro, GI147211 has demonstrated substantial

*Received 18 April 1997*
*Revised 4 March 1998*
*Accepted 16 March 1998*

*Correspondence to:* J O'Dwyer

**Present address:* [1]Servicio de Oncologia Medica, Hospital '12 de Octubre', Carretera de Andalucia KM 5.4, Madrid 28041, Spain

*[4]Present address:* Department of Oncology, Polwarth Building, University of Aberdeen, Forsterhill, Aberdeen AB9 2ZD, UK

Presented in part at the American Society of Clinical Oncology Meeting, May 1995, Los Angeles, California.

activity against a broad range of cell lines. In vivo studies using colon (HT29 and SW480), breast (MX1), ovarian (SKOV3), prostate (PC3) and lung (H460) carcinoma xenografts in nude mice confirmed the anti-tumour activity (Emerson et al, 1986, 1993, 1994, 1995). In preclinical models, topoisomerase I inhibitors demonstrate greater in vitro and in vivo anti-tumour activity when administered by repeat doses or by prolonged infusion. To exploit this apparent schedule dependency, we initiated a phase I trial of GI147211 by 72-h infusion.

### PATIENTS AND METHODS

### Patient selection

This phase I study was conducted as an international collaborative trial between Fox Chase Cancer Center and the Cancer Research Campaign (CRC) Department of Medical Oncology at the University of Glasgow. Eligible patients had histologically documented solid tumours which were considered to be refractory to conventional therapy. They were over 18 years of age, with an Eastern Cooperative Oncology Group (ECOG) performance status of 0–2. They had adequate bone marrow (WBC count ≥4000 $\mu l^{-1}$, granulocyte ≥2000 $\mu l^{-1}$ and platelet count ≥100 000 $\mu l^{-1}$), liver [bilirubin level ≤1.5 mg $dl^{-1}$ and aspartate aminotransferase (AST) ≤4× upper normal value] and kidney (serum creatinine concentration ≤1.5 mg $dl^{-1}$ or creatinine clearance ≥60 ml $min^{-1}$) function. Patients were required to have recovered from all toxicities of prior treatment and to have received no cytotoxic chemotherapy within the previous 3 weeks (6 weeks in the case of nitrosoureas or mitomycin C). All patients gave written informed consent. During the course of this study, placement of a central vein catheter was made a requirement.

Before therapy a medical history, physical examination, complete blood count, biochemical profile, urinalysis, electrocardiography and chest radiography were performed. Patients were monitored with weekly blood counts and biochemical profiles, and clinical examinations were performed on every course. Doses were not escalated within patients. Results are reported using the Common Toxicity Criteria (Cancer Therapy Evaluation Program, National Cancer Institute, Bethesda, MD, 1988). Patients with measurable disease were evaluated (usually by radiographic scan or X-ray) every other course; those with stable disease or better were continued on therapy. Response criteria were standard (Miller et al, 1981).

**Table 1** Characteristics of treated patients

| | |
|---|---|
| Patients, number | 44 |
| Median age, years (range) | 60 (22–79) |
| Men/Women | 22/22 |
| Performance status | |
| 0 | 21 |
| 1 | 22 |
| 2 | 1 |
| Primary tumour | |
| Colorectal | 23 |
| Ovarian | 5 |
| Renal | 3 |
| Sarcoma | 1 |
| Breast | 2 |
| Pancreas | 2 |
| Head and neck | 2 |
| Others | 6 |
| Prior treatment | |
| None | 4 |
| Chemotherapy alone | 26 |
| Chemotherapy + radiotherapy | 14 |

### Drug administration

Initial stability studies revealed that GI147211 was stable either as a concentrate or as a more dilute solution in 5% dextrose (average pH 5). A uniform preparation of 5% dextrose USP was provided as the sole acceptable diluent for this trial. After dilution in 96 ml, the drug was administered as a continuous infusion using an ambulatory infusion pump. Medication bags and extension tubing were changed every day during therapy and were protected from light at all times. Aliquots of the administered solution were obtained to confirm the stability of GI147211.

Significant interspecies differences in toxicity prompted selection of a starting dose of GI147211 of 0.25 mg $m^{-2}$ $day^{-1}$, which was less than 1/30 the murine $LD_{10}$. GI147211 was administered as a 72-h infusion; courses were repeated every 4 weeks, provided

**Table 2** Haematological toxicity expressed in CTC grades as the worst experienced per patient

| Dose (mg $m^{-2}$ $day^{-1}$) | *n* | Leucopenia | | | | Neutropenia | | | | Thrombocytopenia | | | | Anaemia | | | |
|---|---|---|---|---|---|---|---|---|---|---|---|---|---|---|---|---|---|
| | | 1 | 2 | 3 | 4 | 1 | 2 | 3 | 4 | 1 | 2 | 3 | 4 | 1 | 2 | 3 | 4 |
| 0.25 | 6 | – | – | – | – | – | – | – | – | 1 | – | – | – | 2 | – | – | – |
| 0.50 | 4 | 1 | – | – | – | – | – | – | – | – | – | – | – | 1 | – | – | – |
| 1.0 | 5 | – | 1 | 1 | – | 1 | – | – | – | 1 | – | – | 1 | – | – | 1 | – |
| *Minimal pretreatment* | | | | | | | | | | | | | | | | | |
| 1.5 | 5 | – | 2 | – | 1 | – | – | 2 | 1 | 1 | 1 | 1 | 1 | – | 2 | 2 | – |
| 1.75 | 3 | – | – | 1 | – | – | – | 1 | – | – | – | – | – | 1 | – | 1 | – |
| 2.0 | 10 | 4 | 1 | 1 | 2 | 2 | 2 | – | 3 | 2 | 1 | 4 | – | 4 | 4 | 1 | – |
| 2.5 | 2 | – | – | 1 | 1 | – | – | – | 2 | – | – | 1 | 1 | – | – | 1 | – |
| *Heavy pretreatment* | | | | | | | | | | | | | | | | | |
| 1.2 | 3 | 1 | 1 | – | – | 1 | – | – | – | 2 | – | 1 | – | 1 | 2 | – | – |
| 1.5 | 4 | – | – | 2 | 1 | – | – | – | 3 | – | – | – | 3 | 1 | 2 | – | – |
| 2.0 | 1 | – | – | – | 1 | – | – | – | 1 | – | – | – | 1 | – | – | 1 | – |

© Cancer Research Campaign 1998

GI147211C

**Figure 1** Structure of GI147211

blood counts had recovered to pretreatment levels. No dose escalation in individual patients was undertaken.

### GI147211 pharmacokinetics

### *Pharmacokinetic sampling*

Blood and urine samples for the determination of GI147211 pharmacokinetics were obtained during the first cycle. Blood samples (7 ml) were collected in heparinized tubes before infusion and at 2, 4, 6, 8, 14, 26, 32, 38, 50, 56, 62 and 72 h after starting the infusion; and at 15, 25 and 45 min, and 1, 1.5, 2, 4, 6, 8, 10 and 12 h after the infusion had been completed. Urine was collected during treatment and in timed samples for 24 h after treatment. Whole-blood samples were placed in ice-water immediately after collection, frozen within 30 min and kept at −20°C until analysis.

### *Analytical procedure*

A GLP-validated HPLC assay was used for all GI147211 measurements (Stafford and St Claire, 1995). GI147211 is extracted from cold blood by a mixture of 1:4 (v/v) acetonitrile and butylchloride. Samples are kept in an ice slurry bath before extraction as the conversion of lactone to carboxylate is slowed down dramatically by the low temperatures of the ice slurry. The organic phase is collected and evaporated down under a stream of nitrogen. The residue is reconstituted with a 1:4 (v/v) solution of acetronitrile and sodium phosphate buffer (pH 4). This solution is injected onto an HPLC system equipped with a BDS Hyperfil $C_8$ column (250 mm × 4.6 mm) and fluorescence detector. The range of the assay is 0.15–100 ng $ml^{-1}$ with sufficient precision and accuracy (coeffecient of variation < 10%). The mobile phase consists of 25% acetonitrile and 10% ammonium acetate buffer pumped at 1.7 ml $min^{-1}$. The internal standard used in this assay is 6,7-dimethoxy-4-methylcoumarin (Stafford and St Claire, 1995).

### *Pharmacokinetic analysis*

Plasma GI147211 concentration vs time curves were evaluated using model-independent methods (Gibaldi, 1984). The average steady-state concentration ($C_{ss}$) was determined by taking the average concentration after steady-state was achieved. Systemic blood clearance (CL) was estimated using the equation:

$$CL = K_0 / C_{ss}$$

where $K_0$ is the infusion rate. The terminal rate constant $\lambda_z$ was determined by linear regression of log transformation of the blood concentration vs time curve following the end of the infusion. The terminal half-life ($t_{1/2}$) was calculated by the equation:

$$t_{1/2} = 0.693/\lambda_z.$$

Sigma Plot (release 2.0, Jandel Scientific, San Rafael, CA, USA) was used to generate plots and perform linear regression of $C_{ss}$ vs

**Table 3** Non-haematological toxicity expressed in CTC grades as the worst experienced per patient

| Dose ($mg\ m^{-2}\ day^{-1}$) | *n* | Nausea | | | | Vomiting | | | | Diarrhoea | | | | Anorexia | | | | Fatigue | | | | Mucositis | | | | Alopecia | | | | Headache | | | |
|---|---|---|---|---|---|---|---|---|---|---|---|---|---|---|---|---|---|---|---|---|---|---|---|---|---|---|---|---|---|---|---|---|---|
| | | 1 | 2 | 3 | 4 | 1 | 2 | 3 | 4 | 1 | 2 | 3 | 4 | 1 | 2 | 3 | 4 | 1 | 2 | 3 | 4 | 1 | 2 | 3 | 4 | 1 | 2 | 3 | 4 | 1 | 2 | 3 | 4 |
| 0.25 | 6 | 3 | – | – | – | – | 1 | – | – | 1 | – | – | – | 2 | – | – | – | – | – | – | – | 1 | – | – | – | – | – | – | – | – | – | – | – |
| 0.50 | 4 | 1 | 2 | 1 | – | 2 | 1 | – | – | 1 | – | – | – | 1 | 2 | – | – | – | – | – | – | – | – | – | – | – | – | – | – | 2 | – | – | – |
| 1.0 | 5 | 3 | 1 | – | – | 3 | – | – | – | 1 | – | – | – | 2 | – | – | – | 4 | – | – | – | – | – | – | – | 1 | – | – | – | 3 | – | – | – |
| 1.2 | 3 | – | 1 | 1 | – | – | 1 | – | – | – | – | – | – | – | – | – | – | 1 | – | 1 | – | – | – | – | – | – | – | – | – | – | – | – | – |
| 1.5 | 10 | 4 | 3 | – | – | 3 | 2 | – | – | – | – | – | – | 5 | 1 | – | – | 2 | 4 | – | – | 2 | – | – | – | 6 | 1 | – | – | 3 | – | – | – |
| 1.75 | 3 | 2 | – | – | – | – | – | – | – | 1 | – | – | – | – | – | – | – | – | – | – | – | – | – | – | – | 1 | – | – | – | – | – | – | – |
| 2.0 | 11 | 4 | 1 | 1 | – | 1 | 2 | 1 | – | 2 | – | 1 | – | 2 | 1 | – | – | 4 | 2 | – | – | 1 | – | – | – | 4 | 2 | – | – | – | – | – | – |
| 2.5 | 2 | 1 | – | – | – | – | – | – | – | 1 | – | – | – | 1 | – | – | – | 1 | – | – | – | 1 | – | 1 | – | – | – | – | – | – | – | – | – |

**Table 4** Pharmacokinetic parameters of GI147211 (lactone) following 72-h continuous infusion

| Dose (mg m$^{-2}$ day$^{-1}$) | $n$ | $C_{ss}$ (ng ml$^{-1}$) Mean ± s.d. % CV | Half-life (h) Mean ± s.d. % CV | Clearance (ml min$^{-1}$ m$^{-2}$) Mean ± s.d. % CV |
|---|---|---|---|---|
| 0.25 | 6 | 0.25 ± 0.10 | ND– | 776 ± 276 |
| | | 39 | | 36 |
| 0.5 | 4 | 0.44 ± 0.14 | ND– | 864 ± 333 |
| | | 33 | | 39 |
| 1.0 | 5 | 0.86 ± 0.15 | 7.9[a] ± 1.4 | 826 ± 139 |
| | | 17 | 18 | 17 |
| 1.2 | 3 | 0.74 ± 0.30 | 6.1[b] ± – | 1279 ± 559 |
| | | 41 | – | 44 |
| 1.5 | 10 | 1.09 ± 0.34 | 7.4 ± 4.3 | 1046 ± 315 |
| | | 31 | 58 | 30 |
| 1.75 | 3 | 1.10 ± 0.23 | 8.9[c] ± 1.2 | 1138 ± 259 |
| | | 21 | 13 | 23 |
| 2.0 | 11 | 1.09 ± 0.24 | 7.2[c] ± 3.0 | 1290 ± 246 |
| | | 22 | 42 | 19 |
| 2.5 | 2 | 1.21 ± 0.55 | 8.5 ± 4.9 | 1554 ± 691 |
| | | 44 | 58 | 44 |
| Overall mean | | | 7.5 | 1074 |
| s.d. | | | 3.2 | 0.363 |
| % CV | | | 43 | 34 |
| $n$ | | | 29 | 44 |

ND = not done: insufficient measurable concentrations. [a]$n$ = 4. [b]$n$ = 1. [c]$n$ =. [d]$n$ = 10.

**Figure 2** Relationship between dose and lactone $C_{ss}$ (patient average value)

dose. Biopak (release 2.0. Portland. OR. USA) was used to estimate $\lambda_z$. PCNONLIN (release 4.0. Apex. NC. USA) was used to fit the pharmacokinetic and pharmacodynamic data to a sigmoid $E_{max}$ (Hill) equation of the following form:

$$\% \text{ decrease in neutrophils or platelets} = \frac{(100 \times C_{ss}^{\gamma})}{(C_{ss}^{\gamma} + EC_{50}^{\gamma})}.$$

### RESULTS

The demographic characteristics of patients treated in this study are presented in Table 1. Forty-four patients received 124 courses of GI147211. The median number of courses per patient was three (range 1–8). A majority of patients had colorectal cancer and the mean performance status was excellent.

### Haematological toxicity

Eight dose levels ranging from 0.25 to 2.5 mg m$^{-2}$ day$^{-1}$ were studied. Myelosuppression was observed at all dose levels and was dose dependent and non-cumulative (Table 2). Only grade I or II haematological toxicity was noted at doses of 1.0 mg m$^{-2}$ day$^{-1}$. with the exception of a single patient who had previously experienced severe myelotoxicity following treatment with carboplatin and taxotere. At doses ≥ 1.5 mg m$^{-2}$ day$^{-1}$. grade IV myelosuppression was observed and criteria for dose-limiting toxicity fulfilled. The severity of myelosuppression seemed to be related to the extent of prior therapy: accordingly. a representative sample of both heavily and minimally pretreated patients was accrued as stipulated previously.

Patients who were heavily pretreated experienced more myelosuppression at a given dose level (Table 2). Four of ten patients treated at 1.5 mg m$^{-2}$ day$^{-1}$ were heavily pretreated: three developed grade IV thrombocytopenia along with grade III or IV neutropenia. Two of these patients developed neutropenic fever in the first cycle. As three patients had DLT episodes. the next dose level selected for heavily pretreated patients was 1.2 mg m$^{-2}$ day$^{-1}$. Three patients were treated at this dose level without significant bone marrow toxicity. However. before the effect of previous treatment on haematological toxicity became apparent. one heavily pretreated patient was included at 2.0 mg m$^{-2}$ day$^{-1}$: he developed grade IV neutropenia and thrombocytopenia. Therefore. in heavily pretreated patients. the MTD was 1.2 mg m$^{-2}$ day$^{-1}$. At this dose. only minimal neutropenia was observed. Platelet nadirs appeared 18 days after the beginning of the infusion (range 15–22) with a median count of 75 × 10$^9$ l$^{-1}$ (range 31–93).

In contrast to the heavily pretreated patients. two of six minimally pretreated patients entered at 1.5 mg m$^{-2}$ day$^{-1}$ had transient grade III and one grade IV neutropenia. Two patients experienced grade III or worse thrombocytopenia. but no episodes of DLT were recorded. At 1.75 mg m$^{-2}$ day$^{-1}$. only one of the three patients experienced grade III neutropenia. Among ten minimally pretreated patients studied at 2.0 mg m$^{-2}$ day$^{-1}$. four experienced grade III/IV myelosuppression. only one of whom met the criteria of DLT. Two additional patients were studied at 2.5 mg m$^{-2}$ day$^{-1}$ and both developed dose-limiting neutropenia during the first course. The MTD for minimally pretreated patients was 2.0 mg m$^{-2}$ day$^{-1}$. At this dose level. the lowest nadir neutrophil count per patient occurred at a median of 16.5 days (range 10–22) after the initiation of the infusion with a median value of 1.59 × 10$^9$ l$^{-1}$ (range 0.02–3.92). The platelet nadir occurred at day 15 (range 8–20) and had a median value of 72 × 10$^9$ l$^{-1}$ (range 7–219).

Grade II or III anaemia occurred in 15 of 29 patients treated at doses ≥ 1.2 mg m$^{-2}$ day$^{-1}$. Nine patients received 13 transfusions of 24 packs of red cells. 67% of them during the first cycle. The median duration of grade III or IV thrombocytopenia was 4 days (range 1–10 days) and five patients required transfusions of platelets (range 1–3). Five of the ten patients with grade IV

© Cancer Research Campaign 1998

**Figure 3** Relationship between dose and lactone clearance. An increase in clearance is shown at doses > 1 mg m$^{-2}$ day$^{-1}$

neutropenia episodes (median duration 5 days, range 1–14) developed fever and two had microbiologically documented infections.

### Non-haematological toxicity

Twenty-six patients experienced grade I or II nausea and 16 had grade I or II vomiting (Table 3). Three patients had grade III vomiting. Fifteen of these patients had nausea and/or vomiting before starting the infusion of GI147211. Emesis was not dose dependent, usually started on day 1 or 2 and pursued an intermittent course during the infusion. It was easily controlled with standard antimetics. Mild diarrhoea occurred in seven patients and did not appear to be temporally related to treatment. Half of the patients had mild anorexia and/or fatigue which lasted for approximately 2–3 days after the infusion. Alopecia, observed in 14 patients, occurred most frequently in patients who received three or more courses at doses ≥ 1.5 mg m$^{-2}$ day$^{-1}$. Twenty per cent of patients complained of headache while receiving the infusion. In one patient, the infusion was discontinued on the third course as she developed sensitivity to the drug (cutaneous rash, respiratory difficulty, eosinophilia). These symptoms first appeared about 10 days after the initial infusion and became progressively more severe with subsequent cycles. The patient's metastatic colorectal cancer was stable through this period. Two patients presented with a new diagnosis of a bleeding duodenal ulcer and oesophageal varices, shortly after entry into the trial.

Five patients treated at doses < 1.0 mg m$^{-2}$ day$^{-1}$ had the GI147211 infusions administered through peripheral veins. All of these patients developed phlebitis at the site of venepuncture, frequently requiring a change of the i.v. cannula at 24–48 h. Phlebitis recovered in 7–24 days without apparent effect of the applied local treatment. One patient had extravasation of drug in the left forearm: this did not produce necrosis and was successfully managed with local steroids and ice packs. The other 39 patients were treated through central venous catheters. Five developed associated infections requiring i.v. antibiotics. The only toxic

**Figure 4** Relationship between the percentage of reduction in neutrophils (**A**) and platelets (**B**) vs the lactose $C_{ss}$. The curves represent the fit of the data as described in the text death in this study occurred in a non-neutropenic patient who developed staphylococcal septicaemia in association with a Hickman line infection. Three patients had their central vein devices removed, including one who developed a subclavian venous thrombosis.

© Cancer Research Campaign 1998

### Pharmacokinetics

All 44 patients had blood sampling performed during the first course of GI147211. Derived lactone pharmacokinetic parameters are shown in Table 4. There were no significant differences in drug elimination between the two sites. Steady-state GI147211 blood concentrations were reached in most patients between 14 and 26 h into the 72-h infusion. Linear regression analysis revealed that the GI147211 lactone $C_{SS}$ increased with dose over the range 0.25–1.24 ng ml$^{-1}$ ($r^2 = 0.58$, $P < 0.05$) (Figure 2). After the end of infusion, the plasma lactone elimination was monoexponential with a mean half-life of 7.5 h. Mean clearance values tended to increase with dose ($r^2 = 0.31$; $P < 0.05$) and ranged from 776 to 1554 ml min$^{-1}$ m$^{-2}$ (average 1074 ml min$^{-1}$ m$^{-2}$) over the dose range studied (Figure 3).

Total GI147211 blood concentrations (lactone plus carboxylate) were approximately four times greater than lactone concentrations. The variability of total drug was greater than that of the lactone (the active moiety). Terminal half-life of total GI147211 was longer than that of the lactone: the harmonic mean value was 20.1 ± 34.5 h. The clearance of the carboxylate averaged 300 ± 111 ml min$^{-1}$ m$^{-2}$ and also showed a trend to increase with dose. Examination of individual curves indicates that the 'steady-state' concentrations tend to creep up slowly, consistent with the long half-life. These increases in concentration are, however, minor.

Total GI147211 urine concentrations are available from 15 subjects. The mean fraction of drug excreted unchanged in the urine ($f_e$) was 0.114 ± 0.041 and the renal clearance averaged 164 ± 61 ml min$^{-1}$ across the doses measured.

### Pharmacodynamics

The $C_{SS}$ of the GI147211 lactone correlated with the decrease in neutrophil and platelet counts using the sigmoid $E_{max}$ model (Figure 4A and B). While variability was high, a steep concentration–response curve was observed between 0.5 and 1.5 ng ml$^{-1}$. Based on this model, neutrophils were more sensitive than platelets to GI147211 toxicity. The half-maximal concentration was 0.75 ng ml$^{-1}$ for the former and 1.4 ng ml$^{-1}$ for the latter.

### Anti-tumour activity

Three partial responses lasting 8, 30 and 34+ weeks were observed. One patient with breast cancer, who had received two previous chemotherapy regimens (CMF and epirubicin) as well as two prior regimens of hormonal therapy, experienced shrinkage of cutaneous and lymph node disease. A patient with ovarian cancer who had been treated with two platinum-based regimens experienced a substantial decrease in CA125 and had control of ascites. A patient with colorectal cancer metastatic to the liver, previously treated with 5-fluorouracil and leucovorin, also had a partial response. Two additional patients, one with colorectal cancer and the other with hepatoma, had decreases in hepatic lesions of 44% and 36% respectively. All of these patients were treated at doses ≥ 1.5 mg m$^{-2}$ day$^{-1}$, with the exception of the patient with ovarian carcinoma, who received 0.5 mg m$^{-2}$ day$^{-1}$.

### DISCUSSION

Treatment with GI147211 administered as a 72-h continuous infusion in adults with malignant solid tumours was well tolerated on an outpatient basis. As in studies of GI147211 given daily for 5 days, myelosuppression was the dose-limiting toxicity (Eckardt et al, 1995). In the current trial, bone marrow toxicity appeared at all dose levels and involved all haematopoietic lineages in a dose-dependent manner. The degree of platelet toxicity was somewhat more pronounced than that observed on a similar schedule with topotecan (Burris et al, 1994), though more protracted infusions of that drug result in comparable effects on both lineages (Hochster et al, 1994). The extent of prior therapy has also been a determinant of bone marrow toxicity in the 5-day study of GI147211 and in phase I trials of other topoisomerase I inhibitors (Grochow et al, 1992; Saltz et al, 1993; Haas et al, 1994; Eckardt et al, 1995; Rubin et al, 1995). Both neutropenia and thromobocytopenia were dose limiting in this study and both appeared to be related to the extent of previous treatment, although the patient numbers in each population did not allow for rigorous statistical analysis. In heavily pretreated patients, grade III or IV toxicity was observed in all patients treated at or above 1.5 mg m$^{-2}$ day$^{-1}$. Thus, the recommended dose for phase II trials for previously treated patients is 1.2 mg m$^{-2}$ day$^{-1}$ Among untreated and minimally pretreated patients, the MTD was 2.0 mg m$^{-2}$ day$^{-1}$. At this dose, three of ten patients developed grade IV neutropenia and four grade III thrombocytopenia. All of these episodes were uncomplicated and were of short duration (less than 5 days), apart from one case of neutropenic fever. Therefore, an appropriate phase II dose would be 1.75 mg m$^{-2}$ day$^{-1}$.

While the ability of the bone marrow to recover from GI147211 is an important factor, it may be observed from Table 2 that prior treatment alone is insufficient to account for the wide variability in toxicity at a particular dose level (see, for example, the 2 mg m$^{-2}$ day$^{-1}$ dose level). Nor do differences in drug exposure explain the variability: as may be observed in Figure 4, a broad range of toxicity outlines the characteristic sigmoidal curve. The steep concentration–response curve is characteristic of this class of drug (Grochow et al, 1992; Haas et al, 1994) and emphasizes the need to understand the pharmacodynamic basis of drug effect. In vitro studies of cell lines with varying topoisomerase I content suggest that the expression of topoisomerase I may be a determinant of camptothecin effect: the higher the topoisomerase I expression the more sensitive the cell line to camptothecin-induced cytotoxicity (Pommier et al, 1994). However, Pommier et al (1994) have shown that topoisomerase I activity alone does not explain varying sensitivity. In peripheral mononuclear cells from patients undergoing treatment with the topoisomerase I inhibitor topotecan, the opposite relationship has been found: those expressing lower topoisomerase I levels had more myelotoxicity (Khater et al, 1995). Clearly, a simple explanation for the variable toxicity is not yet evident and additional pharmacodynamic studies are needed.

The modest incidence of non-haematological toxicity makes GI147211 an excellent candidate for combination with other drugs or radiation therapy. Mild and easily preventable emesis and short-lasting fatigue were the main complaints. In contrast to irinotecan, GI147211 does not induce diarrhoea (Slichenmyer et al, 1993; Potmesil, 1994; Abigerges et al, 1995). This may relate to the fact that GI147211 is the active compound, while irinotecan is a prodrug which is converted to SN-38, the active metabolite. An important site of this conversion appears to be the intestinal mucosa, in which high levels of SN-38 may be formed locally, with attendant mucosal damage (Gupta et al, 1994). The lack of gastrointestinal toxicity of GI147211 doubtless accounted for the infrequent finding of sepsis, even in the face of profound neutropenia. Also remarkable is the absence of mucositis in the

© Cancer Research Campaign 1998

present study. Only one episode of grade III stomatitis was seen, and this occurred at 2.5 mg m$^{-2}$ day$^{-1}$, concomitantly with neutropenic fever. By contrast, mucositis was dose limiting in a 5-day infusion phase I study of topotecan in leukaemic patients (Kantarjian et al, 1993). The absence of urothelial toxicity is similar to topotecan and is probably due to improved water solubility (Emerson et al, 1995). The propensity of GI147211 to induce phlebitis at venepuncture sites with infusion times of less than 24 h makes the use of central vein catheters a prerequisite for protracted administration schedules.

Anti-tumour activity was documented in five patients with colon, ovary and breast cancers and hepatoma. These three partial and two minor responses are particularly noteworthy as they occurred in patients who had been previously treated with conventional chemotherapy. This, coupled with the significant antiproliferative activity of GI147211 in preclinical models in breast, colon and ovarian tumours (Emerson et al, 1995), and the anti-tumour activity seen with the daily × 5-day regimen, justifies further phase II evaluation GI147211. Except for the partial response in the patient with ovarian cancer observed at 0.5 mg m$^{-2}$ day$^{-1}$, all other tumour regressions appeared at higher dose levels (1.5–2.0 mg m$^{-2}$ day$^{-1}$), suggesting a dose–response relationship. This high rate of response was also identified in another GI147211 phase I trial and these data suggest that the drug may have useful activity when used in a population with less advanced disease.

Pharmacokinetic studies showed that $C_{SS}$ was related to dose but there was a two- to threefold variation in $C_{SS}$ with dose level. $C_{SS}$ at doses ≥ 1.2 mg m$^{-2}$ day$^{-1}$ were above 1 ng ml$^{-1}$ which are potentially cytotoxic (e.g. 50% growth inhibition concentration for the melanoma cell line Lox was 0.592 ng ml$^{-1}$) (Emerson et al, 1995). The $C_{SS}$ of topotecan lactone at MTD of 1.6 mg m$^{-2}$ day$^{-1}$ administered as a continuous infusion for 3 days every 3 weeks was 5.5 ng ml$^{-1}$ (Burris et al, 1994). These differences in drug levels at doses that produce similar degrees of myelosuppression may be accounted for by topotecan being measured from plasma and GI147211 from whole blood, and the observation that the latter compound is 2–5 times more active in in vitro models (Slichenmyer et al, 1993; Emerson et al, 1995). It remains to be seen if this enhanced potency of GI147211 will translate to a selective advantage in clinical trials.

The terminal half-life (7.5 h) in the present study was higher than that observed after 30 min infusions, possibly reflecting the absence of tissue distribution which has already occurred during the 72-h infusion period. Better characterization of the elimination of the carboxylate would be achieved with later sampling points. From these data, the slower clearance of the carboxylate form (mean terminal half-life 20 h) may also provide a source of continuing formation of lactone during the elimination phase. Although greater interpatient variability was observed, the average clearance increased with increasing dose, particularly at doses > 1.0 mg m$^{-2}$ day$^{-1}$. These data do not support a definitive conclusion, but rate-limited elimination or binding processes may be operating at higher doses. As the major route of metabolism of the lactone is its conversion to the open ring acid form, it is unlikely that the compound induces its own metabolism. In this study, approximately 11% of total GI147211 was recovered unaltered in urine, confirming animal data that biliary and/or intestinal excretion are the main routes of elimination for this drug.

In summary, myelosuppression is the DLT of GI147211 as a 72-h infusion every 3 weeks. Haematological toxicity was dose-related, non-cumulative, reversible and dependent on the extent of prior chemotherapy. Central venous catheters are required as prolonged infusions induce phlebitis. At the recommended doses for phase II trials (1.75 mg m$^{-2}$ day$^{-1}$ and 1.2 mg m$^{-2}$ day$^{-1}$ for untreated/minimally treated and heavily pretreated patients respectively), the schedule is well tolerated in an outpatient basis with minimal non-haematological toxicity and promising evidence of anti-tumour activity. The anti-tumour activity documented in this trial supports a broad phase II evaluation of this drug, which is now in progress using the five daily dose schedule. In attempting to optimize the therapeutic efficacy of GI147211, the duration of tumour exposure to the drug may be increased by prolonging the infusion time or by oral administration. For this reason, phase I trials testing 14- and 21-day infusions every 4–5 weeks and a bioavailability study of an oral formulation of the compound have been started.

### ACKNOWLEDGEMENTS

Supported in part by grants from the Cancer Research Campaign, The European School of Medical Oncology, FIS, and CA 06927 from NIH, DHHS and a grant from Glaxo Wellcome. The author acknowledges the expert clinical consistency of Janie McMillan, Janice Graham and Dr Phil Collis.

### REFERENCES

Abigerges D, Chabot GG, Armand JP, et al. Phase I and pharmacologic studies of the camptothecin analog irinotecan administered every 3 weeks in cancer patients. *J Clin Oncol* **13**: 210–221

Burris 3rd HA, Awada A, Kuhn JG, Eckardt JR, Cobb PW, Rinaldi DA, Fields S, Smith L and Von Hoff DD (1994) Phase I and pharmacokinetic studies of topotecan administered as a 72 or 120 h continuous infusion. *Anti-Cancer Drugs* **5**: 394–402

Eckardt JR, Rodriguez GI, Burris HA, et al (1995) A Phase I pharmacokinetic study of the topoisomerase inhibitor GG221 (abstract). *Proc Am Soc Med Oncol* **14**: 476

Emerson DL, McIntyre G, Lucio MJ and Wissel PS (1986) Pre-clinical anti-tumor activity of a novel water-soluble camptothecin analog. *J Med Chem* **29**: 2358–2363

Emerson DL, Vuong A, McIntyre G, Croom DK and Besterman JM (1993) *In vivo* efficacy of two new water-soluble camptothecin analogs in the human cancer xenograft model. *Proc Am Assoc Cancer Res* **34**: 419

Emerson DL, McIntyre G, Lucio MJ, et al (1994) Preclinical antitumor activity of a new water-soluble camptothecin analog (GI147211C). Proceedings of the NCI-EORTC Symposium on New Drugs. *Cancer Ther* **8**: 185

Emerson DL, Besterman JM, Brown HR, et al (1995) *in vivo* antitumor activity of two new seven-substituted water-soluble camptothecin analogues. *Cancer Res* **55**: 603–609

Gibaldi M (1984) *Biopharmaceutics and Clinical Pharmacokinetics*, 3rd edn, pp. 17–28. Lea and Febiger: Philadelphia

Giovanella BC, Stehlin JS, Wall ME, et al (1989) DNA topoisomerase I targeted chemotherapy of human colon cancer in xenografts. *Science* **246**: 1046–1050

Gottlieb JA, Guariano AM, Call JB, et al (1970) Preliminary pharmacologic and clinical evaluation of camptothecin sodium (NSC 100880). *Cancer Chemother Rep* **54**: 461–470

Grochow LB, Rowinsky EK, Johnson R, Ludeman S, Kaufmann SH, McCabe FL, Smith BR, Hurowitz L, DeLisa A and Donehower RC (1992) Pharmacokinetics and pharmacodynamics of topotecan in patients with advanced cancer. *Drug Metab Dispos* **20**: 706–713

Gupta E, Lestingi TM, Mick R, Ramirez J, Vokes EE and Ratain MJ (1994) Metabolic fate of irinotecan in humans: correlation of glucuronidation with diarrhea. *Cancer Res* **54**: 3723–3725

Haas NB, LaCreta FP, Walczak J, Hudes GR, Brennan J, Ozols RF and O'Dwyer PJ (1994) Phase I/pharmacokinetic study of topotecan by 24 hour continuous infusion weekly. *Cancer Res* **54**: 1220–1226

Hochster H, Liebes L, Speyer J, Sorich J, Tanbes B, Oratz R, Wernz J, Chachoua A, Raphael B, Vinci RZ and Blum RH (1994) Phase I trial of low dose continuous topotecan infusion in patients with cancer: an active and well-tolerated regimen. *J Clin Oncol* **12**: 553–559

© Cancer Research Campaign 1998 

Hsiang YH and Liu LF (1988) Identification of mammalian topoisomerase I as an intracellular target of the anticancer drug camptothecin. *Cancer Res* **48**: 1722–1728

Husain I. Mohler JL. Seigler HF and Besterman JM (1994) Elevation of topoisomerase I messenger RNA. protein and catalytic activity in human tumors: demonstration of tumor-type specificity and implicative for cancer chemotherapy. *Cancer Res* **54**: 539–546

Kantarjian HM. Beran M. Ellis A. et al (1993) Phase I study of topotecan. a new topoisomerase I inhibitor. in patients with refractory or relapsed acute leukemia. *Blood* **81**: 1146–1151

Khater C. Yao K-S. Green F. et al (1995) Interindividual variation in topoisomerase I expression and topotecan toxicity. *Proc Am Soc Cancer Res* **86**: 450

Miller AB. Hoogstraten B. Staquet M and Winkler A (1981) Reporting results of cancer treatment. *Cancer* **47**: 207–214

Muggia FM. Creaven PJ. Hanson HH. et al (1972) Phase I clinical trial week and daily treatment with camptothecin (NSC 100880): correlation with preclinical studies. *Cancer Chemother Rep* **56**: 515–521

Pommier Y. Leteurtre F. Fesen MR. Fujimori A. Bertrand R. Solary E. Kohlhagen G and Kohn KW (1994) Cellular determinants of sensitivity and resistance to DNA topoisomerase inhibitors. *Cancer Invest* **12**: 530–542

Potmesil M (1994) Camptothecins: from bench research to hospital wards. *Cancer Res* **54**: 1431–1439

Rubin E. Wood V. Bharti A. et al (1995) A phase I and pharmacokinetic study of a new camptothecin derivative. 9-aminocamptothecin. *Clin Cancer Res* **1**: 267–276

Saltz L. Sirott M. Young C. Tong W. Niedzwiecki D. Tzy-Jyun Y. Tao Y. Trochanowski B. Wright P. Barbosa K. et al (1993) Phase I clinical and pharmacology study of topotecan given daily for 5 consecutive days to patients with advanced solid tumors. with colony stimulating factor. *J Natl Cancer Inst* **85**: 1499–1507

Slichenmyer WJ. Rowinsky EK. Donehover RC and Kaufmann SH (1993) The current status of camptothecin analogues as antitumor agents. *J Natl Cancer Inst* **85**: 271–291

Stafford CG and St. Claire III RL (1995) High-performance liquid chromatographic analysis of the lactone and carboxylate forms of a topoisomerase I inhibitor (the antitumor drug GI147211) in plasma. *J Chromatogr* (B) **663**: 119–126

Wall ME and Wani MC (1995) Camptothecin and taxol: discovery to clinic. Thirteenth Bruce F. Cain Memorial Award Lecture. *Cancer Res* **55**: 753–760

Wall ME. Wani M. Cook CE. Palmer KH. McPhail AT and Sim GA (1966) Plant antitumour agents: the isolation and structure of camptothecin a novel alkaloid of leukemia and tumor inhibitor from camptotheca acuminata. *J Am Chem Soc* **88**: 3888–3890

Zhang H. D'Arpa P and Liu L (1990) A model for tumor cell killing by topoisomerase poisons. *Cancer Cells* **2**: 23–27

*© Cancer Research Campaign 1998*